# The Presence and Anti-HIV-1 Function of Tenascin C in Breast Milk and Genital Fluids

**DOI:** 10.1371/journal.pone.0155261

**Published:** 2016-05-16

**Authors:** Robin G Mansour, Lisa Stamper, Frederick Jaeger, Erin McGuire, Genevieve Fouda, Joshua Amos, Kimberly Barbas, Tomoo Ohashi, S. Munir Alam, Harold Erickson, Sallie R. Permar

**Affiliations:** 1 Duke Human Vaccine Institute, Duke University, Durham, North Carolina, United States of America; 2 Lactation Support Program, Boston Children’s Hospital, Boston, Massachusetts, United States of America; 3 Department of Cell Biology, Duke University, Durham, North Carolina, United States of America; 4 Department of Pediatrics, Duke University, Durham, North Carolina, United States of America; University of British Columbia, CANADA

## Abstract

Tenascin-C (TNC) is a newly identified innate HIV-1-neutralizing protein present in breast milk, yet its presence and potential HIV-inhibitory function in other mucosal fluids is unknown. In this study, we identified TNC as a component of semen and cervical fluid of HIV-1-infected and uninfected individuals, although it is present at a significantly lower concentration and frequency compared to that of colostrum and mature breast milk, potentially due to genital fluid protease degradation. However, TNC was able to neutralize HIV-1 after exposure to low pH, suggesting that TNC could be active at low pH in the vaginal compartment. As mucosal fluids are complex and contain a number of proteins known to interact with the HIV-1 envelope, we further studied the relationship between the concentration of TNC and neutralizing activity in breast milk. The amount of TNC correlated only weakly with the overall innate HIV-1-neutralizing activity of breast milk of uninfected women and negatively correlated with neutralizing activity in milk of HIV-1 infected women, indicating that the amount of TNC in mucosal fluids is not adequate to impede HIV-1 transmission. Moreover, the presence of polyclonal IgG from milk of HIV-1 infected women, but not other HIV-1 envelope-binding milk proteins or monoclonal antibodies, blocked the neutralizing activity of TNC. Finally, as exogenous administration of TNC would be necessary for it to mediate measurable HIV-1 neutralizing activity in mucosal compartments, we established that recombinantly produced TNC has neutralizing activity against transmitted/founder HIV-1 strains that mimic that of purified TNC. Thus, we conclude that endogenous TNC concentration in mucosal fluids is likely inadequate to block HIV-1 transmission to uninfected individuals.

## Introduction

According to the 2014 UNAIDS report, about 2.1 million new HIV infections occurred with over 200,000 being new pediatric infections, approximately half of which are due to transmission via breastfeeding [1]. A highly effective vaccine to prevent mucosal HIV-1 acquisition remains elusive. Thus, development of safe and effective nonvaccine prevention methods is a critical need in the quest to contain the HIV-1 epidemic. Establishing the anti-HIV-1 activities of natural host HIV-1 inhibitors in the setting of the complex mucosal environment is a primary step in achieving the goal of safe and effective nonvaccine prevention methods.

Uninfected breast milk inherently inhibits HIV-1 replication [2–4] and abrogates oral HIV-1 transmission in humanized mice [5]. Several antiviral glycoproteins in breast milk are known to have anti-HIV-1 properties, including lactoferrin [6, 7] and mucin-1 (MUC-1) [8]. Studies have also shown that secretory leukocyte protease inhibitor (SLPI) is another mucosal factor that can inhibit HIV-1 replication [9], but unlike lactoferrin and MUC-1, the anti-viral mechanism of SLPI does not involve direct binding to HIV-1 virions but interaction with the target cells [10]. Recently, Tenascin- C (TNC), a novel HIV-1 inhibitor with neutralizing activity, was identified in the high molecular weight fraction of breast milk [11]. TNC is an extracellular matrix protein previously known to be involved in wound healing and fetal brain development [12, 13]. TNC is a disulfide-linked hexamer where each subunit ranges from 190–300 kDa and is imaged as a symmetrical hexametric structure [14]. TNC binds to the HIV-1 envelope (Env) third variable loop (V3) in the region of the chemokine co-receptor binding site, potentially explaining its ability to block virus infection [11]. Moreover, TNC has broad neutralizing activity against a variety of chronic and transmitted HIV-1 strains and both captures HIV-1 virions and blocks their interaction with mucosal epithelial cells [11]. Studying the kinetics and function of TNC both alone and in concert with other mucosal factors that interact with the HIV-1 Env would contribute to understanding the role of TNC in HIV-1 transmission and its potential to be developed as a safe, novel prophylaxis agent to prevent HIV-1 transmission.

The HIV-1 inhibitory activity of mucosal fluids has been compared across mucosal compartments, with whole saliva and breast milk being the most potently antiviral, followed by seminal fluid and cervicovaginal secretions [3]. Semen has been reported to have both inhibitory and enhancing factors on HIV-1 infection and replication; thus the role of semen in blocking sexual transmission of HIV-1 remains unclear [15]. Specific vaginal HIV-1 inhibitors are not as well studied in the literature. As TNC is a newly identified mucosal HIV-1 neutralizing protein in milk, we sought to determine if it is present and potentially contributes to HIV-1 inhibition in other mucosal compartments that are relevant sites of transmission. Despite the low potency of TNC, exploring the presence of this broad innate mucosal HIV-1 inhibitor and its potential HIV-1 inhibitory role within these complex mucosal fluids is important to defining its potential contribution to HIV-1 transmission *in vivo*.

## Materials and Methods

### Study population

Human breast milk from 18 lactating, U.S. women who tested HIV negative during pregnancy was collected from discarded stores following infant hospitalization at Children’s Hospital Boston after consent of the parent by lactation specialists for donation to research, which was approved by the Harvard Medical School Institutional Review Board. Use of deidentified, discarded human breast milk samples was approved for exemption to protocol review by the Duke University School of Medicine Institutional Review Board (Pro00030437). Donors were selected for having multiple milk samples available from various time points post delivery. Breast milk of HIV-1-infected and uninfected lactating women was collected in the CHAVI009 study of chronically HIV-1-infected, Malawian women at 4 to 6 weeks after delivery [2]. All HIV-1-infected women and infants were provided with single-dose nevirapine during labor if they were not already taking a nevirapine-containing regimen, to prevent mother to child transmission according to the national policy during 2008 to 2009. Semen, cervicovaginal lavage (CVL) fluid, and saliva was collected from both Malawian and U.S. chronically HIV-1-infected and uninfected individuals in the CHAVI001 study [16]. CVL was collected by washing the cervix and ectocervix with 10 ml of normal saline or PBS and collecting the fluid that pooled in the posterior fornix.

### Identification of TNC in semen and CVL

Semen and CVL from 4 uninfected donors each were pooled and centrifuged to remove cells and other debris. Samples were fractionated by using a size exclusion column (Superose 6 10/300 GL; GE Healthcare Life Sciences) with 1X PBS as the running buffer on high performance liquid chromatography (HPLC). Fractions containing the higher molecular weight proteins (>500 kDa) were then separated by charge on a strong anion exchanger (Source 15Q 4.6/100 PE; GE Healthcare Life Sciences) and using a salt gradient as described previously [11]. Presence of TNC was determined by Western blot with an anti-TNC monoclonal antibody (81C6) [17], and confirmed by mass spectrometry using a nanoscale capillary LC/MS/MS system where the protein band was processed and analyzed using data-dependent acquisition as previously described [11], then quantified by TNC ELISA (see below). Potential protease activity in mucosal fluid that could degrade TNC was tested by incubating semen, CVL, and breast milk with recombinant TNC at 37°, room temperature, and 4°C. TNC was quantified by TNC ELISA before and after incubation (see below).

### Quantification of TNC in mucosal fluids

Milk samples were delipidized by centrifugation at 21,000×g. Semen, saliva, and cervical vaginal lavage fluid samples were also centrifuged to remove cells and other debris. To quantify TNC in these mucosal fluids, a 384-well plate was coated with 2 μg/ml of anti-TNC antibody rabbit polyclonal IgG (H-300, Santa Cruz Biotechnology) and incubated at 4°C overnight. Wells were washed with PBS + 0.1% Tween-20 using BioTek ELx405 Microplate washer and blocked with 7.5% Bovine Serum Albumin (Gibco) at room temperature for 1 hour. Samples were diluted in 7.5% BSA and a commercially-available purified TNC (Millipore) was used as the protein standard ranging from 5μg/ml to 5ng/ml. The standards and samples were added to the plate and incubated for 1 hour at room temperature. Plates were washed 2 times and 1 μg/ml of Tenascin-C Antibody mouse monoclonal (T2H5, Fisher) was added and incubated for 1 hour at room temperature. Plates were washed 2 times and a 1:10,000 dilution of goat anti-mouse HRP-conjugated antibody (Promega) was added to each well and incubated for 1 hour at room temperature. Plates were washed 4 times and SureBlue Reserve TMB Microwell Substrate (KPL) was added and incubated in the dark at room temperature for 5 minutes. TMB Stop Solution (KPL) was added and plates were read at 450nm. Detection of TNC in a Western blot was comparably sensitive to TNC ELISA, as the same detection antibody (T2H5, Fisher) was used in both assays, with expected decreasing OD_450_ magnitude consistent with loss of detection of the same concentration of TNC by Western blot.

### Depletion and isolation of IgG from breast milk

IgG was isolated from breast milk using Protein G resin pre-packed into 96-well depletion plates (GE Healthcare). Plasma was diluted 2-fold with TBS, pH 7.5, and 200 μl of the diluted sample was added per well. The plates were incubated at room temperature, with shaking, for one hour. The unbound fractions were removed by centrifugation at 700 x g for 3 minutes. Wells were then washed 3 times with 400 μl of TBS to remove loosely bound material. The IgG bound to the resin was eluted with 200 μl of 2.5% glacial acetic acid, pH 2.53, and immediately neutralized with 120 μl of 1M Tris-HCL pH 9.0.

### Neutralization of HIV-1 by breast milk and breast milk proteins

All neutralization assays were performed as described previously (1, 11) with a few adjustments as described. Milk samples were delipidized as described above and filtered through 0.22μm Spin-X centrifugal filter (Costar). Breast milk is then concentrated 4 times using Amicon Ultra-0.5 mL 30K Centrifugal Filters (Millipore). A starting dilution of 1:2.5, 1:5, or 1:10 of the concentrated breast milk and serial 3-fold dilutions were tested for neutralization potency against HIV-1 clade C virus DU156.12 (tier 2) in TZM-bl cells [18, 19]. All assays were observed under the microscope to check for cell morphology. If observed cellular toxicity exceed the dilution at which 50% neutralization was observed, results were not reported. We previously established that purified TNC was not toxic to the cell line used for neutralization [11], yet breast milk supernatant can demonstrate toxicity. The inhibitory dilution or concentration at which 50% of the virus was neutralized (ID_50_) for each sample was calculated based on the reduction in relative light units (RLUs) compared with virus-only control. To assess the potency and synergy of neutralization of breast milk proteins, neutralization assays were done by combining purified TNC (Millipore) with purified human lactoferrin (Bio-rad), or recombinant MUC-1 (Abcam) at starting concentrations of 200μg/ml, 500μg/ml, and 200μg/ml respectively, followed by serial 3-fold dilutions of the dual protein mixtures.

For HIV-1 neutralization of TNC at different pHs, 1X PBS (Life Technologies) was adjusted to pH = 4, 5, 6 using HCl. TNC Millipore was concentrated 10 fold and buffer exchanged into 1X PBS (pH = 4, 5, 6) using 30K Amicon spin columns. TNC Millipore was incubated in various pH PBS for 1 hour at room temperature. TNC was then buffer exchanged back into 1X PBS (pH = 7.4) prior to addition to the reporter cells to avoid cell toxicity. TNC was used at a starting dilution of 1:5 for neutralization assay as previously described [2,11].

For HIV-1 neutralization of TNC in the presence of other anti-HIV-1 antibodies, we incubated purified IgG from HIV-1 positive and negative breast milk with virus before adding to serially diluted purified TNC (Millipore). To test HIV-1 neutralization of TNC in the presence of a V3-specific antibody, CH22, at 100 μg/ml was incubated with HIV-1 clade C virus DU156.12 (tier 2) prior to addition of serially diluted purified TNC. CH65, an anti-hemagglutinin monoclonal antibody, was used at the same concentration as CH22 as a negative control. To test HIV-1 neutralization of a broadly neutralizing anti-HIV-1 antibody, we use PG9 at a sub-neutralizing concentration (0.1 μg/ml) in a similar manner as described when testing the V3 specific antibody. Synagis was used at the same concentration as PG9 (0.1 μg/ml) as a negative control. To test if the presence of TNC affected broadly neutralizing anti-HIV-1 antibodies, TNC at neutralizing concentration (200 μg/ml) was incubated with HIV-1 clade C virus DU156.12 (tier 2) before addition to serially diluted PG9 starting at 50 μg/ml. Synagis was also serially diluted in the same manner as PG9 as a negative control.

### HIV-1 Env variable 3 (V3) loop binding by IgG from HIV-1 infected breast milk

384-well plates were coated with 30ng of HIV-1 MN.V3 peptide at 4°C overnight. The plate was then blocked for 1hr at room temperature with Super Block (4% whey, 15% goat serum, 0.05% Tween-20, 80.5% 1X PBS). CHAVI009 HIV-1 breast milk samples, 4–6 weeks postpartum, were delipidized by centrifugation as described above. Breast milk samples were three fold serially diluted in Super Block twelve times, starting at 1:3 dilution. The V3-specific antibody CH22 was used as the standard starting at 50 ug/ml and 3-fold serially diluted. V3 binding was detected using anti-Human IgG HRP diluted at 1:5000 in Super Block. Sure Blue TMB Microwell Peroxidase Substrate (KPL) and TMB Stop Solution (KPL) were used to develop the ELISA. Plate was read at 450nm on SpectroMax plate reader.

### Measurement of the total protein and other identified HIV-1 inhibitors concentration in breast milk and other mucosal fluids

Delipidized milk and centrifuged semen and CVL were used in a Bradford assay to determine total protein content, according to the manufacturer’s directions (BioRad). Briefly, 5μl of sample was mixed with 250ul of Coomasie dye (BioRad) in a 96-well flat bottom plate. The dye and sample were mixed and allowed to incubate for a minimum of 5 minutes before being read at 595nm. Bovine Serum Albumin (BSA) ranging from 125 μg/ml to 2000 μg/ml was used as the protein standard. Lactoferrin in breast milk was measured using a human lactoferrin ELISA kit (Innovative Research). Breast milk samples were diluted in PBS ranging from 1:50,000 to 1:100,000 and lactoferrin was measured as detailed by the manufacturer. Human lactoferrin provided in the kit ranging from 0ng/ml to 100ng/ml was used as a standard control. Mucin-1 (MUC-1) in breast milk was measured using ELISA Kit for MUC-1 (Cloud-Clone Corp). MUC-1 ranging from 0.156 ng/ml to 100ng/ml was used as a standard. Breast milk samples were diluted in PBS ranging from undiluted to 1:1000 and MUC-1 was measured as suggested by the manufacturer.

### Production of recombinant TNC

Recombinant full-length TNC (rTNC) was made by transfecting 293T Lenti-X cells with pEE14-HxB.L using jetPRIME transfection reagent (Polyplus Transfection). Cells were incubated in Hybridoma Serum Free Media (Life Technologies) at 37°C for 6 days. Cell supernatant was filtered through a 0.22 μm filter to remove cell debris. Solid ammonium sulfate was added to the cell supernatant to 35% saturation at 4°C and centrifuged to pellet precipitate. The pellet was resuspended in 1XPBS and separated by high performance liquid chromatography (HPLC) using a size exclusion column (Superose 6 30/100 GL; GE Healthcare Life Sciences) equilibrated in 1X PBS buffer. TNC-containing fractions were collected and concentrated using 30K Amicon centrifugal filters (EMD Millipore). Purified TNC was quantified using TNC ELISA and tested against HIV-1 viruses B.YU.2, C.MW965, C.BF1677, C.BF1677, C.BF329NFU2, C.BF942, C.1086, C.DU156.12, and B.MN.3 in a neutralization assay described above.

### Statistical analysis

The nonparametric Spearman correlation coefficient was used to determine the correlation of TNC, lactoferrin, MUC-1, total protein, and V3-specific antibody concentration with HIV-1 neutralizing potency (ID_50_) of mucosal fluid samples. A Mann-Whitney rank test was used to compare TNC concentrations between HIV-1-infected and uninfected mucosal fluids and between mucosal compartments. All statistical tests were performed with Graphpad (La Jolla, CA) Prism version 6.

## Results

### Identification of TNC in genital mucosal fluids

**Fig 1:**
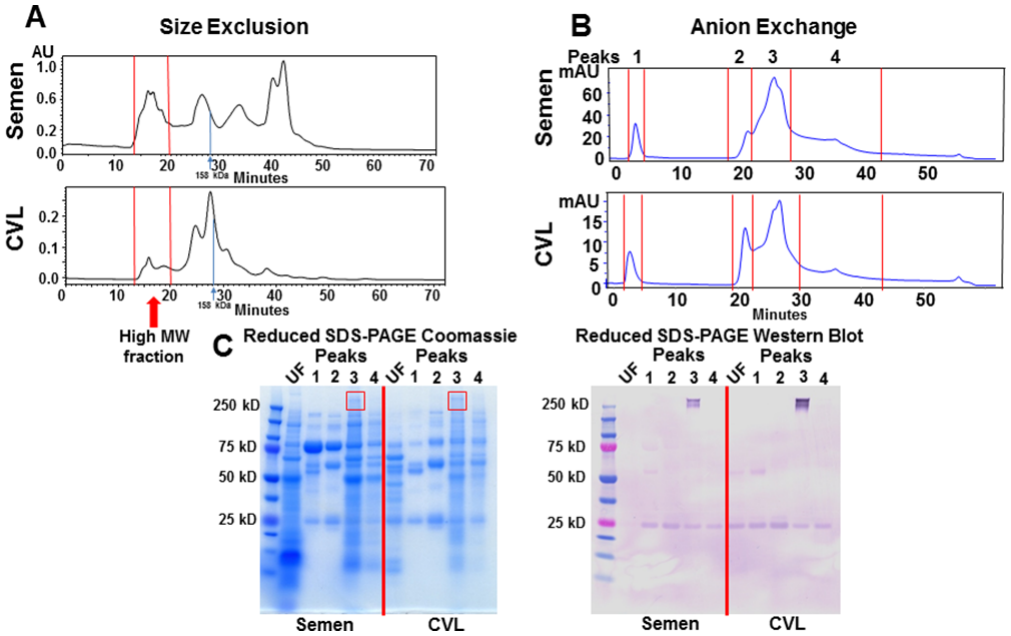
Identification TNC in semen and CVL. (A) Pooled semen and CVL was separated by size (>500 kDa) and charge (B) by high performance liquid chromatography (HPLC). (C) Unfractionated (UF, diluted 1:10) and charge-separated, high molecular weight (MW) semen and CVL protein peaks were then run on a reduced SDS-PAGE gel and Coomassie stained. TNC was detected with an anti-TNC monoclonal antibody. The 250 kD band staining on anti-TNC western blot was confirmed to be TNC by LC/MS.

To explore the presence of TNC at mucosal compartments other than breast milk, we first screened for the presence of TNC in various mucosal fluids by TNC ELISA. TNC was detected above the minimum detection level of 0.005 μg/ml in 12/40 semen and 11/63 CVL samples, but only 1/31 saliva samples by the TNC ELISA. Samples that had undetectable levels of TNC were shown at minimum level of detection. To confirm the presence of TNC in semen and vaginal fluid, we performed a similar TNC isolation method to that previously applied to breast milk [11]. We fractionated pooled semen and CVL of uninfected individuals by size exclusion and collected the high molecular weight protein pool (Fig 1A). We then further fractionated the high molecular weight protein fraction on the basis of charge by anion exchange, resulting in four main peaks for both semen and CVL (Fig 1B) and ran these fractions on a reduced SDS-PAGE coomassie gel. A band that appeared at 250kDa in both semen and CVL matched the size of reduced TNC monomers and, in fact, was detected by an anti-TNC monoclonal antibody by western blot in both semen and CVL (Fig 1C). The appearance of the band as a doublet could either be due to a cleavage product, or the presence of the TNC-short (TNC-S) isoform [14]. TNC was detected by a monoclonal antibody (81C6) that is specific to the fibronectin domain which is present in both long and short forms of TNC. Finally, the presence of TNC in these CVL and semen protein fractions were confirmed by nanoscale capillary LC/MS/MS system mass spectrometry of the 250kDa band and comparison of identified peptides to a human protein database [11].

**Fig 2:**
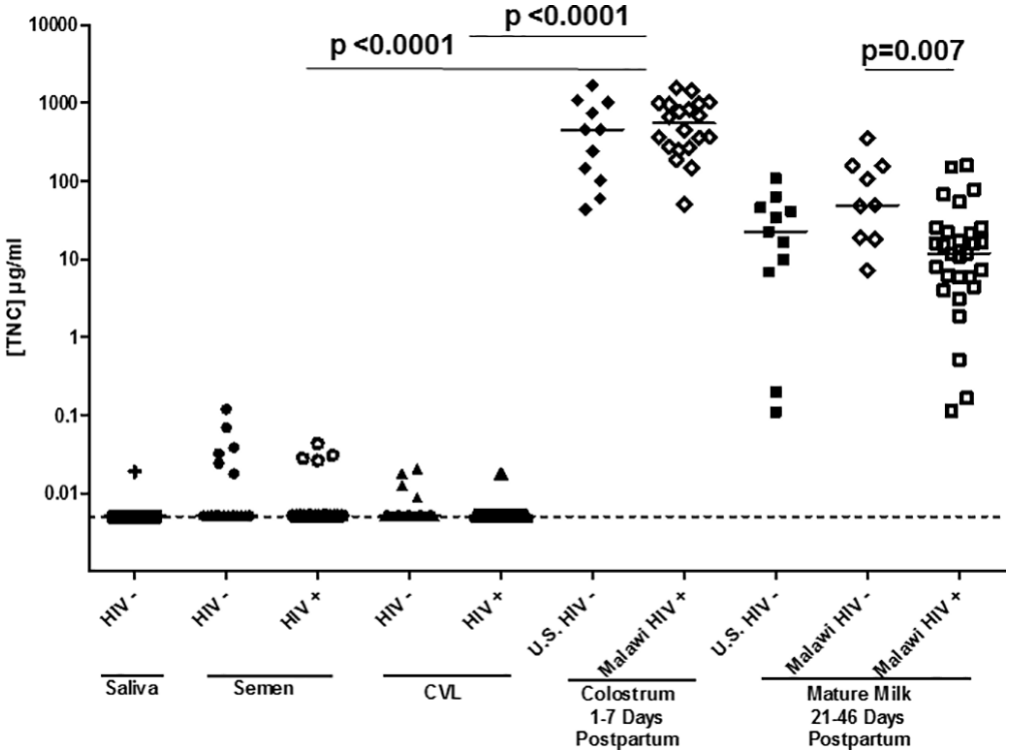
TNC is infrequently detected and more than 2 logs lower in concentration in semen and CVL compared to milk, yet the concentration and detection frequency is similar in HIV-1-infected and uninfected individuals. TNC concentration was measured via ELISA in saliva, semen, CVL, colostrum, and mature milk for both HIV-1 infected (open symbols) and uninfected (closed symbols) individuals. The concentration of TNC in colostrum was significantly higher than that in CVL, semen, or mature milk in both HIV-infected or uninfected individuals. There was no significant difference between TNC levels in breast milk of uninfected women from the U.S. and Malwai. The minimum detection level of 5 ng/ml is indicated by the dotted line.

We next compared the concentration of TNC determined by ELISA in these mucosal fluids of HIV-1-infected and uninfected subjects. The median concentration of TNC was considerably lower in CVL fluid (median = 0.005 μg/ml detected in 11/63 samples, 52 samples were below detection level of 4.88 ng/ml) and semen (median = 0.029 μg/ml detected in 12/40 samples, 28 samples were below detection level of 4.88 ng/ml) than in mature milk (median = 18.11 μg/ml, p < 0.001) (Fig 2). There was no significant difference between TNC concentration between HIV-1 positive and HIV-1 negative mucosal fluids for each compartment (Mann-Whitney U test). There was no significant difference in the mature milk TNC concentration between HIV-1 uninfected U.S. and Malawian women. However, the median milk TNC concentration was slightly higher in uninfected (median = 49.1 μg/ml) Malawian women compared to HIV-1 infected (median = 11.9 μg/ml) Malawian women (Mann-Whitney U test, p = 0.007). TNC concentration was a median of 1.44% (range: 0.06–6.14%) of total breast milk protein concentration, yet only 0.0044% (range: 0.0004–0.01%) of the total protein concentration in semen (p<0.0001) (Table 1). As genital fluids have considerable protease activity, we next tested the susceptibility of recombinant TNC to *ex vivo* degradation in the presence of semen and CVL. Interestingly, there was considerable degradation of recombinant TNC after incubation with semen and CVL overnight at 37°C compared to breast milk (Table 2). Therefore, the concentration and rate of detection of TNC in genital fluids may be severely underestimated *ex vivo*.

**Table 1:** The concentration of TNC compared to total protein in breast milk, semen, and CVL of HIV uninfected individuals.

Sample Type	[Total Protein] μg/ml	[TNC] μg/ml	% TNC
Breast milk	1310.93	33.56	2.56
Breast milk	1047.71	1.13	0.11
Breast milk	1113.90	1.86	0.17
Breast milk	1111.34	22.56	2.03
Breast milk	1034.50	18.11	1.75
Breast milk	1047.27	13.28	1.27
Breast milk	1266.78	77.74	6.14
Breast milk	940.89	3.19	0.34
Breast milk	1007.76	7.30	0.72
Breast milk	1144.88	5.26	0.46
Breast milk	1367.28	124.01	9.07
Breast milk	1157.93	18.73	1.62
Breast milk	895.06	0.51	0.06
Breast milk	1329.50	36.70	2.76
Semen	873.09	0.04	0.00
Semen	1263.90	0.07	0.01
Semen	1252.74	0.12	0.01
Semen	772.12	0.03	0.00
Semen	1135.31	0.06	0.01
Semen	1025.17	0.04	0.00
Semen	1208.51	0.01	0.00
CVL	48.16	0.01	0.02
CVL	<125 μg/ml	<0.005 μg/ml	N/A
CVL	<125 μg/ml	<0.005 μg/ml	N/A
CVL	<125 μg/ml	<0.005 μg/ml	N/A
CVL	<125 μg/ml	<0.005 μg/ml	N/A
CVL	<125 μg/ml	<0.005 μg/ml	N/A
CVL	<125 μg/ml	<0.005 μg/ml	N/A
CVL	<125 μg/ml	<0.005 μg/ml	N/A
CVL	<125 μg/ml	<0.005 μg/ml	N/A
CVL	<125 μg/ml	<0.005 μg/ml	N/A
CVL	<125 μg/ml	<0.005 μg/ml	N/A

**Table 2:** TNC concentration before and after incubation in semen, CVL, and breast milk.

Mucosal Sample	Temperature	TNC (μg)	% Reduction
Semen Only	N/A	0.00	N/A
CVL Only	N/A	0.00	N/A
Breast milk Only	N/A	3.96	N/A
TNC Only	N/A	121.16	N/A
Semen	37°C	0.62	96.65
CVL	37°C	0.02	99.91
Breast milk	37°C	16.82	8.62
PBS	37°C	18.41	N/A
Semen	RT	3.65	84.02
CVL	RT	0.42	98.15
Breast milk	RT	23.94	-4.84
PBS	RT	22.84	N/A

### TNC neutralization activity after low pH treatment and in the presence of other anti-HIV innate factors in breast milk

**Fig 3:**
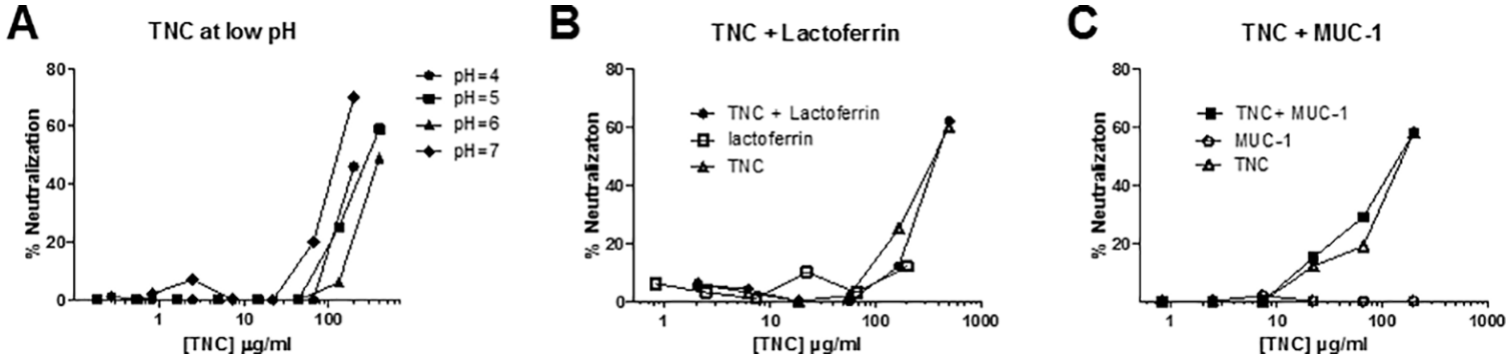
Antiviral activity of TNC in low pH conditions and in the presence of other mucosal innate HIV-1 inhibitors. (A) Neutralization potency of purified TNC was measured after low pH treatment to simulate the low pH of the vaginal compartment against HIV-1 C.DU156. (B) Neutralization potency of purified TNC starting at 200 μg/ml, purified lactoferrin starting at 500 μg/ml, and TNC plus lactoferrin (reported in TNC concentration) was determined against HIV-1 C.DU156 in TZM-bl cells. (C) In a separate assay, purified TNC starting at 200 μg/ml, recombinant MUC-1 starting at 200 μg/ml, and TNC plus MUC-1 (reported in TNC concentration) was determined against HIV-1 C.DU156. Neither the addition of purified lactoferrin or MUC-1 to TNC had additive or inhibitory effects on TNC’s HIV-1 neutralizing potency.

Genital fluids are a unique environment in that vaginal fluid is characteristically low pH (range 2.8–8.0), while semen is typically neutral or high pH (range 7.2–8.0) [20, 21, 22]. As TNC was identified as a component of semen and vaginal fluid, we investigated whether low pH treatment impacted the ability of TNC to neutralize HIV-1. After treatment at pH 4, 5, 6, and 7, TNC was able to mediate similar HIV-1 neutralization potency (Fig 3A) with an inhibitory concentration of 50% neutralization of 200, 298.5, 400, 128.5 μg/ml, respectively. This indicates that TNC could be active against HIV-1 in vaginal mucosa despite exposure to the low pH environment with potency 2–4 fold lower than that at neutral pH.

Since purified TNC can neutralize HIV-1 *in vitro*, yet TNC was not well-correlated to neutralizing potency of milk of HIV-infected or uninfected women, we reasoned that other milk proteins in breast milk with the ability to bind HIV-1 virions may serve to enhance or inhibit breast milk neutralizing capacity of TNC. Thus, we studied the impact of the presence of other HIV-1-neutralizing inhibitors, lactoferrin and MUC-1, on the *in vitro* HIV-1 neutralization potency of TNC. We selected starting concentrations of lactoferrin and MUC-1 based on the average concentration of these proteins in human milk and performed serial dilutions in tandem with TNC. The TNC neutralization curve was unchanged in the presence of either protein (Fig 3B and 3C), indicating that these innate antiviral proteins neither inhibit nor synergize with the neutralization activity of TNC at the concentrations tested.

### The relationship between TNC concentration and the HIV-1 neutralizing activity of breast milk of uninfected women over time in the postpartum period

**Fig 4:**
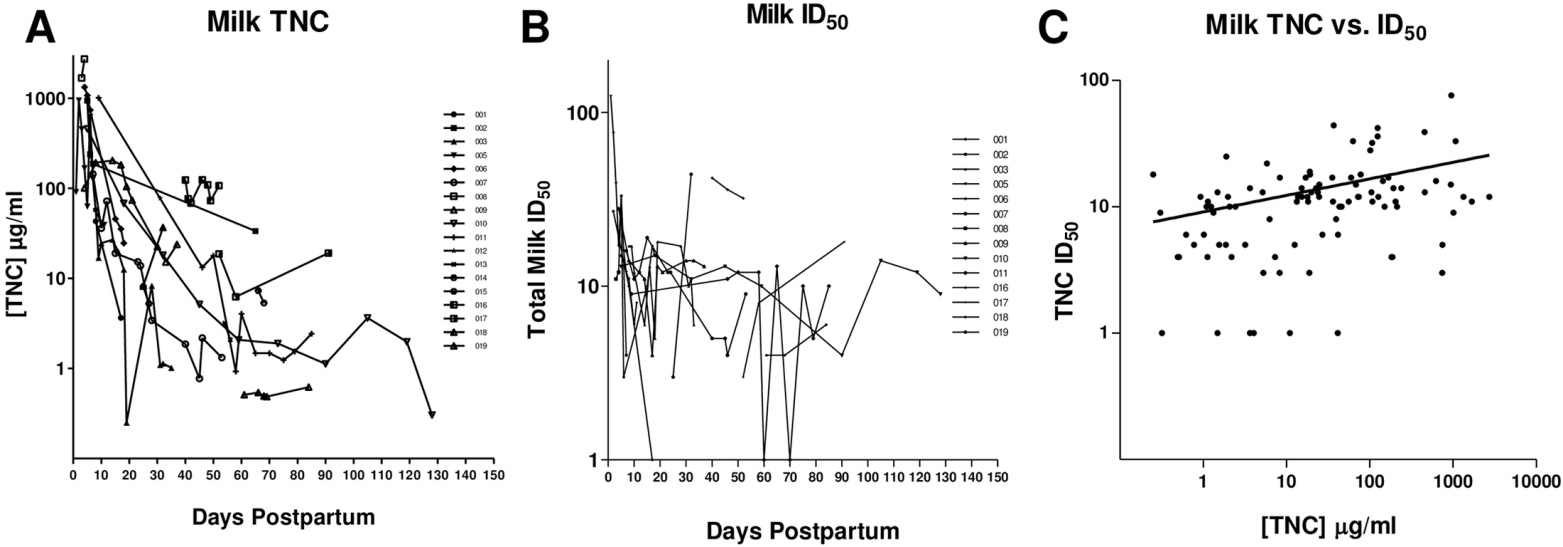
The Tenascin-C concentration and HIV-1 neutralizing potency of breast milk decreases over the postpartum period. (A) The amount of TNC in milk was measured by ELISA over time in 18 uninfected lactating women. Each line represents a distinct lactating woman. (B) Innate HIV-1, neutralization activity for samples from each lactating women was measured in a neutralization assay against HIV-1 variant C.DU156 in TZM-bl cells and reported in 50% inhibitory dilution (ID_50_). (C) Spearman’s rank correlation of TNC concentration and ID_50_ against HIV-1 C.DU156 for all the uninfected milk samples revealed a weak direct correlation by Spearman’s rank test (r = 0.38, p = 0.0001).

While a limited quantity of TNC was found in semen and CVL, breast milk contains a considerably higher concentration, providing a mucosal fluid that can be used to more easily examine the HIV-1 inhibitory role of this protein in the setting of a complex, protein-rich fluid. To first determine how the concentration of TNC in milk changes over time postpartum, we measured the TNC concentration in milk of 18 uninfected women collected at multiple time points post delivery. In the early lactation period, the TNC concentration drops greater than10-fold with a median of 239.58 μg/ml in milk collected less than 10 days postpartum (range: 16.9 to 2727 μg/ml) compared to a median 3.41μg/ml in milk collected at greater than 30 days postpartum (range: 0.3 to 211 μg/ml) (Fig 4A) (p = 0.0041, Mann Whitney-U). In contrast, the innate HIV-1 neutralization potency of uninfected breast milk against the HIV-1 variant C.DU156 (chronic clade C virus with tier 2 neutralization sensitivity) [23], measured as inhibitory dilution 50% (ID_50_), only slightly declined during the early postpartum period with a median of 14.5 at less than10 days postpartum (range: 3 to124) and a median ID_50_ of 10 at greater than 30 days postpartum (range: <3 to 44) (Fig 4B). ID_50_, as opposed to TNC IC_50_, is reported here because total breast milk neutralization measures the composite effect of all components of breast milk. By 30 days postpartum, TNC concentration was below the reported *in vitro* HIV-1 inhibitory concentration 50% (IC_50_) of TNC (range: 109–158 μg/ml) (11) in all women in this cohort.

In correlating the TNC concentration and neutralization potency in milk collected at all time points from the 18 women, TNC concentration from uninfected breast milk only demonstrated a weak direct correlation with the ID_50_ against C.DU156 (r = 0.38, p = 0.0001) (Fig 4C). Upon further stratification and using a single time point per patient, there was no correlation between TNC concentration and HIV-1 neutralization in milk collected at less than 2 weeks or 2–4 weeks postpartum (Table 3). However, there was a direct correlation between TNC concentration and HIV-1 neutralization activity in mature milk (greater than 4 weeks postpartum) of uninfected women (r = 0.65, p = 0.006). Yet, a similar correlation between neutralization and concentration was also observed for other anti-HIV factors in breast milk with reported neutralizing activity, including lactoferrin (r = 0.58, p = 0.02) and MUC-1 (r = 0.77, p = 0.05), as well as a trend towards a correlation with total milk protein concentration (r = 0.50, p = 0.07) (Table 3).

**Table 3:** Spearman’s rank correlations of the concentration of TNC and anti-HIV activity in mature milk of HIV infected and uninfected women.

**Breast milk of uninfected women**		
[TNC] vs. ID_50_, 0–2wks	r = -0.31	p = 0.36
Virus: C.DU156.12 (tier 2)		
[TNC] vs. ID_50_, 2–4wks	r = 0.40	p = 0.25
Virus: C.DU156.12 (tier 2)		
**[TNC] vs. ID**_**50**_, **> 4wks**	**r = 0.65**	**p = 0.006**
**Virus: C.DU156.12 (tier 2)**		
**[Lactoferrin] vs. ID**_**50**_, **> 4wks**	**r = 0.58**	**p = 0.02**
**Virus: C.DU156.12 (tier 2)**		
**[MUC-1] vs. ID**_**50**_, **> 4wks**	**r = 0.84**	**p = 0.002**
**Virus: C.DU156.12 (tier 2)**		
Total Protein Concentration vs. ID_50_, > 4wks	r = 0.50	p = 0.07
Virus: C.DU156.12 (tier 2)		
**Breast Milk of HIV-infected women**		
[TNC] and milk viral load	r = 0.189	p = 0.334
[TNC] and Na/K ratio (marker for mastitis)	r = -0.002	p = 0.993
[TNC] and neutralization ID_50_	r = 0.162	p = 0.403
Virus: C.MW965.26 (tier 1)		
**[TNC] and neutralization ID**_**50**_	**r = -0.412**	**p = 0.026**
**Virus: C.DU156.12 (tier 2)**		
[TNC] and neutralization ID_50_	r = -0.154	p = 0.426
Virus: C.CAP.45.2.00.G3 (tier 2)		
**[TNC] and neutralization ID**_**50**_	**r = -0.389**	**p = 0.037**
**Virus: C.DU422.1 (tier 2)**		

**Fig 5:**
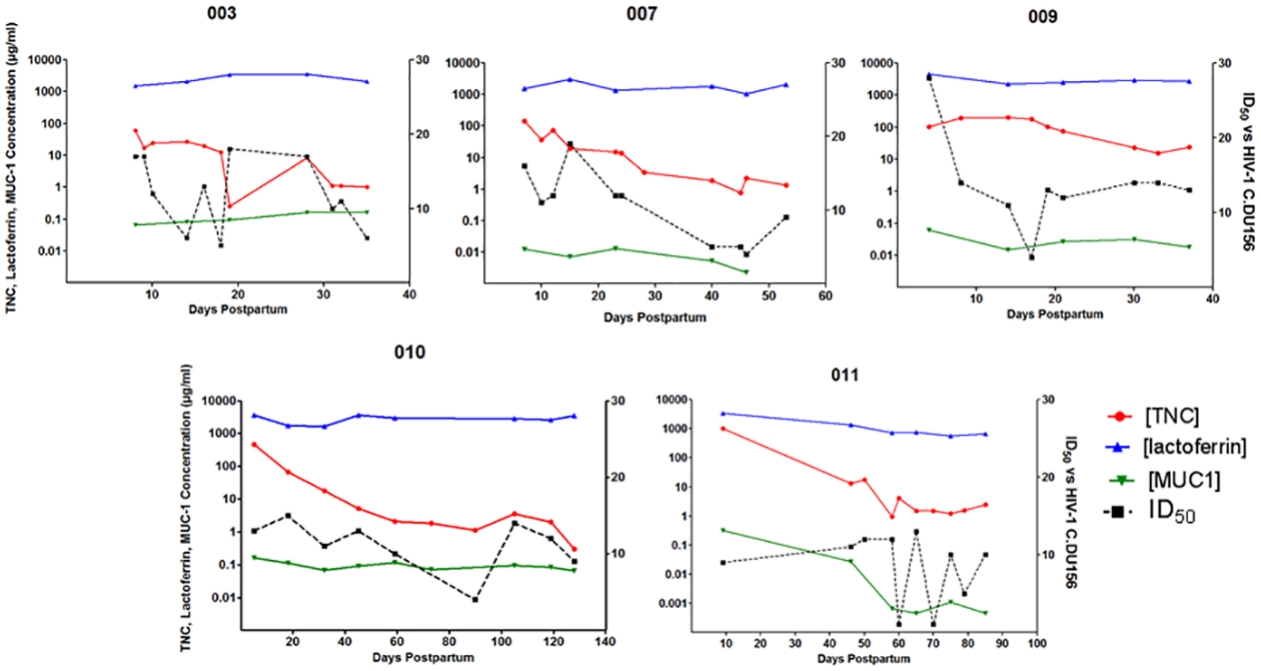
Kinetics of the concentration of innate milk HIV-1 inhibitors lactoferrin, TNC, and MUC-1 and the innate HIV-1 neutralizing potency of individual uninfected women over the postpartum period. TNC, lactoferrin, and MUC-1 concentrations in milk were determined via ELISA and plotted over the postpartum period for uninfected, lactating women. HIV-1 neutralizing potency (ID_50_) of milk was measured by neutralization assay against HIV-1 variant C.DU156 in TZM-bl cells over time postpartum. Each graph represents milk from an individual uninfected woman over the postpartum period.

To observe the time course of HIV-1 neutralizing activity and the concentration of HIV-1 neutralizing proteins in milk in more detail, we plotted these values over the period of lactation for 5 uninfected women. In general, lactoferrin was the most abundant of the three proteins while MUC-1 had the lowest concentration. Concentrations of lactoferrin and MUC-1 in milk were stable over time postpartum for each woman, whereas TNC concentration and the HIV-1 neutralizing potency of milk (ID_50_) varied over time, though not always congruently. Therefore the concentration of any one identified HIV-1 neutralizing protein does not appear to predict the innate HIV-1 neutralizing activity of whole milk (Fig 5).

### Relationship between TNC concentration, HIV-1 virus load, breast inflammation, and neutralization potency in breast milk of HIV-1 infected, lactating women

Breast milk virus load and inflammation are two previously identified breast milk factors that influence postnatal HIV-1 transmission risk [24]. Thus, we sought to define the relationship between TNC, milk virus load, and breast inflammation (mastitis), and HIV-1 neutralization potency of milk of HIV-1-infected women. No correlation was found between TNC concentration and virus load or sodium/potassium ratio (a marker for mastitis) in milk collected at 4–6 weeks postpartum from HIV-1 infected women [25]. Moreover, no direct correlation was found between TNC concentration and neutralizing activity against several clade C HIV-1 variants including C.MW965.12 (tier 1), C.DU156.12 (tier 2), C.DU422.1 (tier 2), and C.CAP.45.2.00.G3 (tier 2) in milk of HIV-1-infected women. In fact, an indirect correlation was observed between TNC concentration and ID_50_ against HIV C.DU156 and C.DU422 in the milk of HIV-1 infected women (Table 3). We reasoned that the HIV Env-specific IgG in milk of HIV-1-infected women may primarily mediate the neutralization activity, as the adaptive HIV-1 neutralization response in milk has been tied to HIV-1 Env-specific IgG [11]. Moreover, these IgG antibodies, especially those directed against the V3 loop, may block the activity of TNC against HIV-1. Thus, we depleted IgG from milk of HIV-1-infected women to determine the relationship between neutralization potency and TNC concentration in IgG depleted milk of HIV-1-infected women. In fact, the negative correlation between ID_50_ and TNC concentration was abolished in the IgG depleted milk (Table 3).

### Evaluation of inhibitory or synergistic HIV-1 neutralization activity of TNC in the presence of anti-HIV-1 antibodies

**Fig 6:**
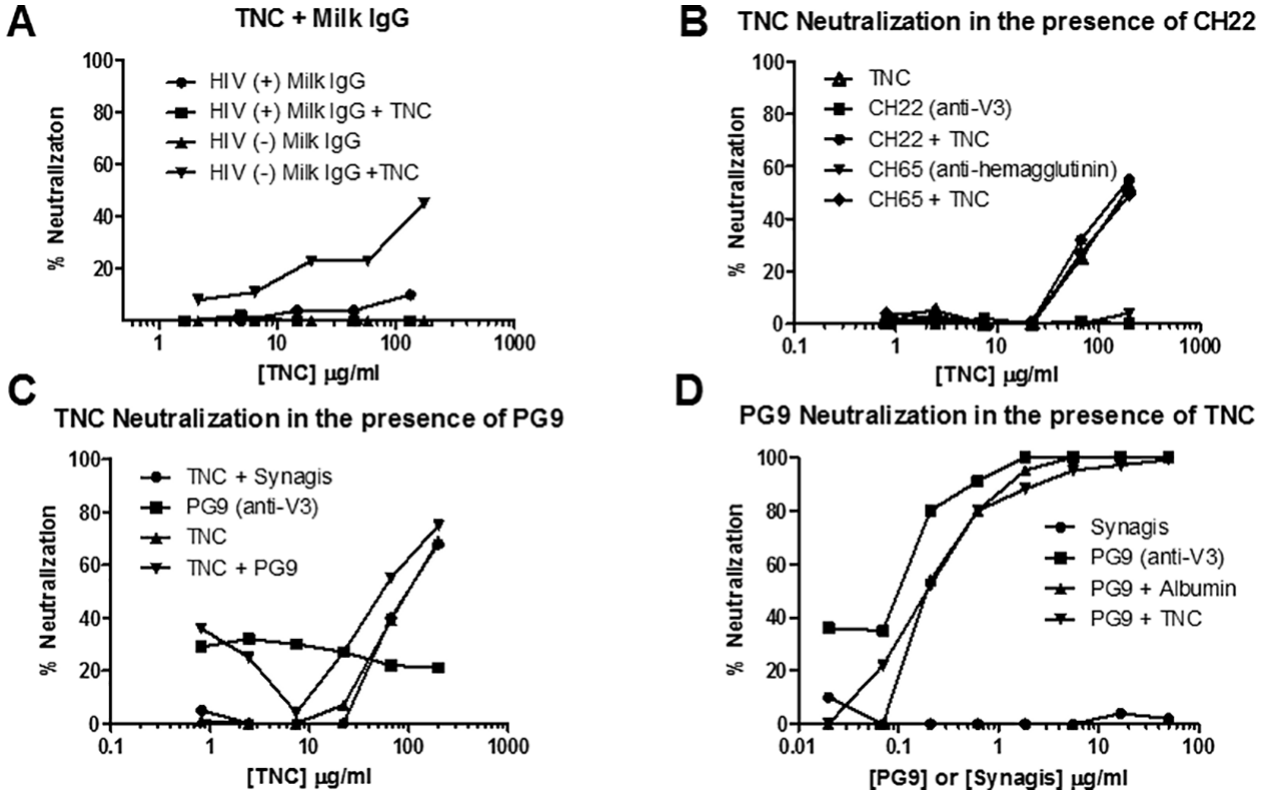
HIV-1 neutralizing activity of purified TNC in the presence of anti-HIV-1 antibodies. (A) IgG from breast milk of HIV-infected women inhibited TNC neutralization against HIV-1 C.DU156 whereas breast milk IgG from uninfected women did not. (B) The presence of either a control (CH65, anti-hemagglutinin) or anti-V3 antibody (CH22, 100 μg/ml) did not block neutralization of C.DU156 by TNC. (C) The presence of broadly neutralizing V3-specific monoclonal antibody PG9 at subneutralizing concentration (0.1 μg/ml) did not inhibit neutralization of C.DU156 by TNC. Synagis, the anti-RSV mAb at 0.1 μg/ml was used as a negative control. (D) The presence of TNC at 200 μg/ml also did not inhibit the neutralization activity of PG9 against HIV-1 C.DU156. Albumin at 200 μg/ml was used as a negative control for TNC and synagis at 50 μg/ml was used as a negative control for PG9.

Due to the negative correlation of TNC and neutralizing potency that we observed in the milk of HIV-1-infected women, but not IgG depleted milk, we reasoned the milk IgG directed against the HIV Env, particularly the V3 loop, may be interfering with TNC’s neutralizing activity. Neutralization by TNC was assessed in the presence of breast milk IgG from HIV-1 infected and uninfected women at a starting concentration similar to that in milk of HIV-1-infected women [11]. In fact, TNC had similar neutralization potency in the presence and absence of IgG from uninfected breast milk, whereas no neutralization by TNC was detected in the presence of IgG from HIV infected breast milk, suggesting the IgG in the breast milk of HIV-1 infected women could interfere with TNC mediated HIV-1 neutralization (Fig 6A). Yet, similar reduction of TNC’s neutralizing activity was not seen in the presences of a monoclonal antibody directed against the known Env epitope binding site of TNC, the V3 loop (CH22) (Fig 6B). Therefore, blocking of TNC’s neutralizing activity may require a polyclonal anti-Env antibody response.

Because TNC binds to the V3 loop of gp120, we assessed if there was neutralization interference of TNC in the presence other V3-specific HIV-1 neutralizing antibodies [11]. Conversely, we also tested for interference of V3-specific HIV-1 neutralization in the presence of TNC. HIV-1 virus C.DU156 was incubated with subneutralizing levels of PG9 (0.1 μg/ml), a broadly neutralizing glycan-dependent V3-specific monoclonal antibody, and neutralization of TNC was measured. Neutralization of PG9 in the presence of TNC was also measured by first incubating the virus C.DU156 with neutralizing levels of TNC (200 μg/ml) and assessing PG9 neutralization potency. At these subneutralizing concentrations, neither TNC and PG9 interfered with neutralization in the presence of the other (Fig 6C and 6D), likely due to the weak interaction between TNC and the V3 loop [11] and slight differences in epitope-specificity between these two molecules [26].

### HIV-1 neutralizing activity of recombinant TNC

**Fig 7:**
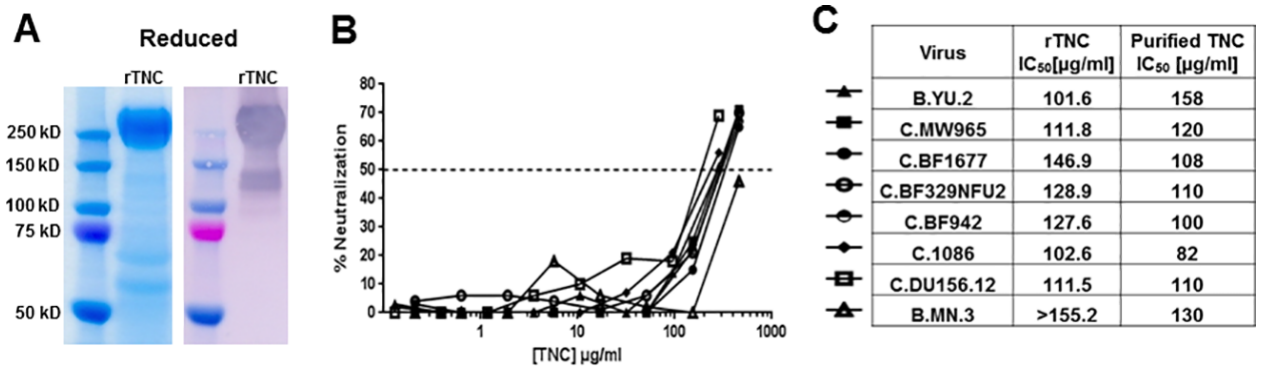
Neutralization potency of recombinant TNC. (A) Recombinant TNC was produced in 293T Lenti-X cells, purified by ammonium sulfate precipitation and HPLC. Purity was observed on a reduced SDS-PAGE gel (Coomassie stained) and a reduced Western blot (anti-TNC stained). (B) The neutralization potency of recombinant TNC was measured against several clade B and C HIV-1 variants as indicated in the table. (C) Recombinant TNC neutralized variants at an IC_50_ of 101.6 μg/ml to >146.9 μg/ml, values comparable to that of purified TNC [11].

As the amount of TNC in breast milk and genital fluid appears to be lower than the amount required for measureable HIV-1 neutralization and can be blocked by polyclonal anti-Env antibodies, application of exogenous TNC in the genital tract or oral administration to infants would be required to harness the HIV-neutralizing effect of TNC. Thus, we sought to produce a recombinant version of TNC that could mediate HIV-1 neutralization, mirroring that of purified TNC. We produced TNC in a LentiX 293 T-cell line and purified via ammonium sulfate precipitation and size fractionation. This TNC, produced in a human 293 T-cell line, demonstrated broad neutralizing activity against multiclade HIV-1 variants (Fig 7) with a potency similar to that observed with purified TNC (median ID_50_ 119.73, range 101.62- >155.20) [11]. Therefore, it is possible to produce a recombinant version of TNC that will mimic the anti-HIV-1 activity of the purified protein from breast milk.

## Discussion

Despite substantial mucosal virus exposure of nursing infants born to HIV-1-infected mothers, in the absence of antiretroviral use, less than 10% acquire HIV-1 via breastfeeding [27]. Similarly, although rates of heterosexual infection of HIV-1 can vary substantially based on several risk factors, one study suggests that risk of infection from heterosexual intercourse could be from 0.04%- 0.55% per exposure [28, 29]. This suggests that there may be innate, passive immune mechanisms protecting mucosal surfaces against HIV-1 acquisition. Defining and understanding these innate antiviral factors may guide strategies to eliminate postnatal and other forms of mucosal HIV-1 transmission. Among those is TNC, an extracellular matrix protein known to be important in fetal development and wound healing, but whose *in vitro* anti-HIV-1 activity was recently discovered [11].

We sought to assess the presence of TNC in other mucosal compartments involved in transmission of HIV-1, so that the extent of its potential *in vivo* antimicrobial activity at mucosal surfaces could be determined. Importantly, the existence of recombinant TNC and TNC in other mucosal compartments was revealed for the first time in this work. While the level of TNC measured in *ex vivo* mucosal fluids does not appear to be impacted by HIV-1 infection status, the TNC concentration is overall very low in these compartments compared to milk due to the possible protease activity found in semen and CVL. Future work should assess the concentration of TNC present in fresh genital fluid samples to better estimate the true *in vivo* concentration of this innate factor. However, even accounting for this degradation, the concentration of TNC in genital fluids does not appear to be high enough to reach neutralizing capacity and therefore likely does not impact sexual HIV-1 transmission. Yet, TNC does remain active after incubation at low pH, consistent with that of the vaginal tract. Since breast milk had the highest concentration of TNC and no detectable degradation, we proceeded to study the kinetics and function of TNC in the compartment where it is most abundant.

TNC concentration in milk correlated weakly with neutralization activity of whole breast milk, which is consistent with its depletion leading to reduced HIV neutralizing activity of whole milk [11]. Yet, it is unclear if TNC is present in a high enough concentration to potentiate HIV-1 neutralization *in vivo*. TNC concentration drops well below the concentration required to neutralize 50% of the virus population *in vitro* TNC neutralization of HIV-1(IC_50_ 109–158 μg/ml) [11] within the first 10 days of lactation and the TNC concentration did not correlate with neutralization in the early milk samples. Although TNC concentration and neutralization activity correlated in milk collected greater than 4 weeks postpartum, so did the concentrations of other milk proteins with reported anti-HIV-1 activity. Moreover, TNC concentration in milk at this time postpartum is below the *in vitro* IC_50_ [11]. Furthermore, there is no correlation between TNC level and neutralization in early milk, when the TNC level *in vivo* would be considered high enough to neutralize HIV-1. This suggests that in this complex mucosal fluid, it may be the physical milieu as a whole that determines the innate capacity of breast milk to mediate HIV-1 neutralization. Yet, when the interaction between TNC and other innate breast milk proteins with reported HIV-1-neutralizing activity, including lactoferrin or MUC-1, was investigated, there was no additive or inhibitory effect on HIV-1 neutralization in the presence of these proteins. Studies of combinations of breast milk proteins and other components, such as carbohydrates and glycopeptides, could more accurately represent the physiological environment of milk, and shed more light on the innate neutralizing capacity of milk and its role in postnatal protection against HIV-1 transmission.

When assessing the relationship between TNC and HIV-1 neutralizing capacity of milk of HIV-1-infected women, there was, in fact, an indirect correlation between TNC concentration and neutralization in mature milk. This could be due to differing amounts of TNC in milk of HIV-1 infected and uninfected women, as we saw slightly higher TNC levels in milk of uninfected Malawian women. Although we did not see a difference in TNC concentration between uninfected Malawian and U.S. women, it is not known if there are distinct milk TNC levels in different HIV-1-infected ethnic populations. We reasoned that HIV-1-infected women have HIV-1 Env-specific antibody in their breast milk [2] which would target the same binding site as TNC, the V3 loop on the gp120 Env protein. The IgG response has been shown to mediate the majority of adaptive virus neutralization in milk and this response may interfere with TNC’s interaction with HIV-1 and vice versa, potentially explaining the lack of direct correlation between TNC concentration and neutralization in milk of HIV-1-infected women. However, when the IgG is depleted from breast milk of HIV-1-infected women there was still no correlation between TNC and neutralization, although, the negative correlation was abolished. TNC may be sterically hindered from interaction with the HIV Env by other proteins in breast milk of HIV-1 infected women, including HIV Env-specific antibodies. In fact, we determined that milk IgG from HIV-1-infected women blocked TNC neutralization *in vitro*, while milk IgG from HIV-1-uninfected women did not. However, the weakly-neutralizing linear V3-specific monoclonal antibody, CH22, did not affect TNC’s neutralizing function suggesting that the blocking of TNC neutralization by milk IgG may be a polyclonal antibody effect. Importantly, TNC did not interfere with neutralization activity of a glycan-dependent broadly neutralizing V3-specific monoclonal antibody, PG9, and vice versa, likely due to affinity and epitope-specificity differences. Thus, exogenous administration of TNC would not be expected to interfere with potent HIV-1 neutralizing antibodies already present in mucosal fluids.

While TNC may not be present in adequate amounts in milk or other mucosal fluids to mediate sufficient HIV-1 neutralization *in vivo*, it consistently demonstrates anti-HIV-1 properties *in vitro*. Thus, upregulation or exogenous administration of TNC would be required for it to be used as an agent to block postnatal or sexual virus transmission. However, the presence of TNC in semen and vaginal fluid suggests that overexpression or exogenous application of this host protein in the genital mucosal compartment should be nontoxic. Moreover, this protein, which is present in high concentration in colostrum, would be uniquely nontoxic for administration to HIV-exposed breastfeeding infants. Importantly, we have shown that a recombinant form of TNC retains the anti-HIV-1 activity of purified TNC. Yet, given the weak neutralizing activity of TNC against HIV-1, its susceptibility to degradation by proteases in genital fluids, and its potential to be blocked by polyclonal anti-HIV antibody in milk, the binding and neutralizing capacity of TNC and other innate factors needs to be further characterized in mucosal fluids. As there remains a need for alternative, safe, and long-lasting methods to prevent both postnatal and sexual HIV-1 transmission, the activity of TNC along with other natural innate HIV-1 inhibitors should be further studied for their antiviral activity to be enhanced and exploited for natural HIV-1 prophylaxis.
